# Total-Arterial Revascularization Is Superior in Heart Failure Patients with Reduced Ejection Fraction—A Propensity Score Matched Retrospective Multicenter Analysis

**DOI:** 10.3390/medsci13030179

**Published:** 2025-09-05

**Authors:** Christian Jörg Rustenbach, Julia Schano, Christoph Salewski, Helene Häberle, Kristian-Christos Ngamsri, Ilija Djordjevic, Stefanie Wendt, Tulio Caldonazo, Ibrahim Saqer, Shekhar Saha, Philipp Schnackenburg, Lina Maria Serna-Higuita, Torsten Doenst, Christian Hagl, Thorsten Wahlers, Christian Schlensak, Stefan Reichert

**Affiliations:** 1 Department of Thoracic and Cardiovascular Surgery, German Cardiac Competence Center, Eberhard-Karls-University of Tuebingen, 72076 Tuebingen, Germany; julia.schano@med.uni-tuebingen.de (J.S.); christoph.salewski@med.uni-tuebingen.de (C.S.); christian.schlensak@med.uni-tuebingen.de (C.S.); stefan.reichert@med.uni-tuebingen.de (S.R.); 2 Department of Anesthesiology and Intensive Care Medicine, Eberhard-Karls-University of Tuebingen, 72076 Tuebingen, Germany; helene.haeberle@med.uni-tuebingen.de (H.H.); kristian.ngamsri@med.uni-tuebingen.de (K.-C.N.); 3 Department of Cardiothoracic Surgery, Heart Center, University of Cologne, 50923 Cologne, Germany; ilija.djordjevic@uk-koeln.de (I.D.); stefanie.wendt@uk-koeln.de (S.W.); thorsten.wahlers@uk-koeln.de (T.W.); 4 Department of Cardiothoracic Surgery, Friedrich-Schiller-University, University Hospital of Jena, 07743 Jena, Germany; tulio.caldonazo@med.uni-jena.de (T.C.); ibrahim.saqer@med.uni-jena.de (I.S.); torsten.doenst@med.uni-jena.de (T.D.); 5 Department of Cardiac Surgery, Ludwig-Maximilians-University, 80539 Munich, Germany; shekhar.saha@med.uni-muenchen.de (S.S.); philipp.schnackenburg@med.uni-muenchen.de (P.S.); christian.hagl@med.uni-muenchen.de (C.H.); 6 German Centre for Cardiovascular Research (DZHK), Partner Site Munich Heart Alliance, 80539 Munich, Germany; 7 Institute for Clinical Epidemiology and Applied Biostatistics, Eberhard-Karls-University of Tuebingen, 72074 Tuebingen, Germany; lina.serna-higuita@med.uni-tuebingen.de

**Keywords:** TAR, heart failure, CABG, OPCAB, HFrEF, low EF

## Abstract

**Background:** Total arterial revascularization (TAR) may improve outcomes in patients with ischemic cardiomyopathy and heart failure with reduced ejection fraction (HFrEF). **Methods:** We retrospectively screened 574 adults with HFrEF (LVEF < 40%) undergoing isolated CABG across four German centers (2017–2023). After 1:1 propensity score matching, 240 patients were analyzed (120 TAR vs. 120 NTAR). The primary endpoint was in-hospital MACCE (death, MI, stroke). Key secondary endpoints included ICU/hospital length-of-stay, ventilation time, delirium, transfusion requirements, and acute kidney injury. **Results:** MACCE occurred in 4.1% (TAR) vs. 14.2% (NTAR) (*p* = 0.007). TAR was associated with shorter ICU stay (median 44.5 h vs. 90 h, *p* < 0.001), shorter hospital stay (10 d vs. 12 d, *p* = 0.002), reduced ventilation time (8 h vs. 12 h, *p* < 0.001), lower delirium (5.0% vs. 14.2%, *p* = 0.016), and fewer RBC transfusions intra-operatively (0.13 ± 0.45 vs. 0.31 ± 0.58 units, *p* = 0.028) and during the entire stay (0.70 ± 1.33 vs. 1.77 ± 2.91 units, *p* < 0.001). **Conclusions:** In this multicenter propensity-matched cohort, TAR was associated with lower in-hospital MACCE and more favorable perioperative outcomes compared with NTAR. Prospective studies are warranted to confirm causality and long-term benefits.

## 1. Introduction

Ischemic cardiomyopathy (ICM), a predominant cause of heart failure with reduced ejection fraction (HFrEF), presents a complex therapeutic challenge, compounded by the intricate interplay of coronary artery disease (CAD) progression and ventricular dysfunction. Coronary artery bypass grafting (CABG) has been advanced as a valid therapy option for ICM management [1]. CABG stands as a cornerstone in the management of ischemic heart disease, especially in the presence of heart failure with reduced ejection fraction (HFrEF) [2]. Within this domain, total arterial revascularization (TAR) has emerged as a promising technique, potentially offering enhanced durability and outcomes compared to traditional revascularization strategies in patients with severe ventricular dysfunction [3]. Despite the burgeoning interest, there remains a paucity of rigorous, multi-center evaluations that delineate the benefits of TAR in a HFrEF cohort following isolated CABG. The evolution of surgical techniques has led to the exploration of TAR as a potentially superior method due to its associated long-term patency rates and survival benefits. Several studies have highlighted the additional advantages of utilizing a second arterial graft, employing either the right internal mammary artery or radial artery [4]. This preference is attributed to their potentially greater long-term patency in comparison to saphenous vein grafts [5]. Despite this, TAR’s efficacy—specifically within the HFrEF subset post-isolated CABG—remains underexplored, particularly on a multicenter scale.

This retrospective, multicenter analysis employs propensity score matching to methodically assess TAR’s efficacy in HFrEF patients, addressing the inherent selection bias in observational studies [6]. By juxtaposing TAR with non-total arterial approaches, this study seeks to distill the impact on postoperative morbidity and mortality, contributing to a nuanced understanding to the global discourse on optimal surgical strategies for HFrEF patients. By evaluating clinical outcomes, this study aims to fill the gap in the literature, providing evidence-based insights to the optimal revascularization technique for enhancing survival in HFrEF patients undergoing isolated CABG. The analysis transcends multi-center observations by integrating data across various demographics and clinical practices, thereby enhancing the generalizability of the findings and reinforcing the evidence base for clinical decision-making.

Given the vulnerability of patients with heart failure and reduced ejection fraction (HFrEF) to perioperative complications, optimizing revascularization strategies remains of paramount importance. Total arterial revascularization (TAR) has the potential to reduce perioperative morbidity by minimizing aortic manipulation, lowering transfusion requirements, and promoting early recovery. We hypothesized that TAR would reduce in-hospital major adverse cardiac and cerebrovascular events (MACCE) and improve short-term postoperative outcomes, including neurocognitive and resource-related endpoints, compared to non-TAR (NTAR) in patients with HFrEF undergoing isolated CABG.

## 2. Materials and Methods

### 2.1. Study Design and Patient Population

The study was conducted in accordance with the Declaration of Helsinki. This project was approved by the IRB of the Tübingen University Hospital with project number (216/2022BO2) from 12 April 2022. 

**Figure 1:**
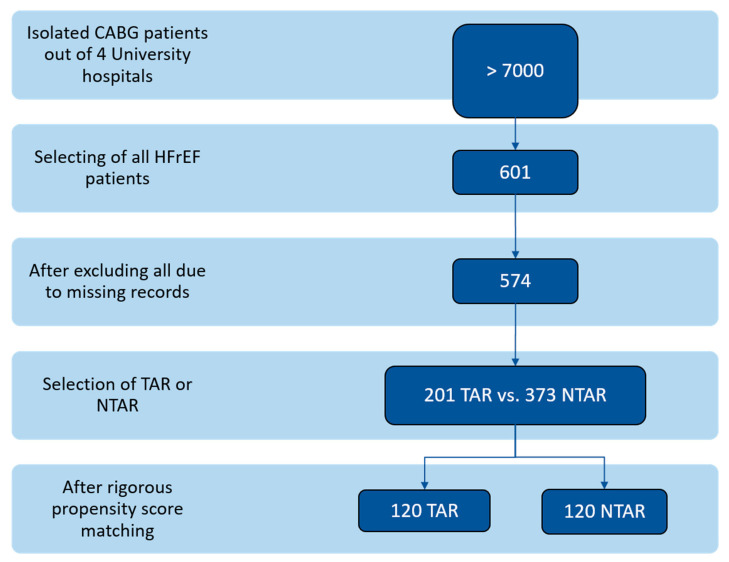
Patient selection flowchart.

This retrospective, multicenter study (2017–2023) analyzed 574 adult patients diagnosed with Heart Failure with reduced Ejection Fraction (HFrEF) who underwent isolated Coronary Artery Bypass Grafting (CABG) at four academic hospitals in Germany (Figure 1). We identified patients with verified left ventricular ejection fraction (LVEF) of less than 40%, presenting with coronary three-vessel disease and/or left main stem stenosis, without the need for cardiac reoperation or those not in cardiogenic shock, preoperative need for resuscitation, catecholamine support, or mechanical ventilation prior to surgery. Incomplete medical records were also excluded. Heart failure with reduced ejection fraction (HFrEF) was defined as symptomatic heart failure with a left ventricular ejection fraction ≤ 40%, according to the current ESC and AHA/ACC Guidelines [7,8]. Patients were then divided into two categories based on the type of grafts utilized: ‘Total Arterial Revascularization’ (TAR) and ‘Non-Total Arterial Revascularization’ (NTAR), employing a propensity score matching (regarding age, gender, etc.) process. This categorization was critical to assess the impact of graft selection on immediate postoperative outcomes, analyzing a comprehensive set of variables, including demographic details, clinical indicators (or risk calculators, respectively) such as EuroScore II and STS Score, health status, cardiac history, intraoperative procedures, and postoperative results.

### 2.2. Primary Outcomes

The primary objective was to assess the occurrence of major adverse cardiac and cerebrovascular events (MACCE), including in-hospital mortality, myocardial infarction (MI), and stroke. Participants were each counted once in the incidence rate, regardless of multiple MACCEs. This methodology enabled the comparison of any significant MACCE occurrence between NTAR and TAR patient groups, focusing on the binary outcome of MACCE presence during hospital stay. Appropriate statistical tests (please see below) were employed to assess the frequency of at least one MACCE event between groups, offering a comprehensive view of the general risk for major adverse events intra- or post-surgery, while also evaluating each event type individually.

### 2.3. Secondary Outcomes

The study further assessed in-hospital events, including acute kidney injury (AKI) with or without necessitating dialysis, delirium (CAM-ICU test in all centers), the need for red blood cell transfusions, duration of intensive care unit (ICU) stay, mechanical ventilation time, total hospital stay, completeness of recascularization, surgical time (incision-to-closure time) and the occurence of sepsis.

### 2.4. Surgical Techniques and Perioperative Care Protocols

All surgeries included in this analysis were performed by senior cardiac surgeons with substantial experience in coronary artery bypass grafting, each with a minimum annual case volume exceeding 50 CABG procedures. This inclusion criterion ensured a consistently high level of surgical proficiency across all participating centers.

A comprehensive description of the surgical approaches, including on-pump (ONCAB) and off-pump (OPCAB) techniques, has been reported previously [9].

In the present study, CABG procedures adhered to international surgical standards. Total arterial revascularization (TAR) was defined as the use of exclusively arterial conduits, including bilateral internal mammary arteries (BIMA) or the left internal mammary artery (LIMA) in combination with the radial artery (RA). Non-total arterial revascularization (NTAR) included at least one venous conduit (saphenous vein graft) in addition to arterial grafts [9].

The choice between TAR and NTAR was based on anatomical suitability, conduit availability, and surgeon preference, as previously described. Within the TAR group, the decision to use BIMA versus LIMA + RA was at the discretion of the surgeon, taking into account patient anatomy, conduit quality, and the individual risk profile for sternal wound complications.

Perioperative management, including anesthesia, ventilation strategies, transfusion thresholds, and intensive care protocols, was standardized according to internal guidelines at each participating institution. Although these institutional guidelines were consistent with current best practice recommendations, minor differences among centers in anesthesia, postoperative sedation protocols, and early extubation practices cannot be entirely excluded and may contribute to variability in postoperative outcomes.

Both on-pump coronary artery bypass (ONCAB) and off-pump coronary artery bypass (OPCAB) procedures were included. Use of cardiopulmonary bypass (CPB) was incorporated as a covariate in the propensity score model. In addition, we performed stratified analyses by pump strategy and tested for a TAR × pump interaction to evaluate whether the effect of total arterial revascularization (TAR) versus non-total arterial revascularization (NTAR) differed according to surgical approach.

Complete revascularization was defined as grafting of all coronary arteries ≥1.5 in diameter with ≥50% stenosis deemed technically suitable by the surgical team, consistent with prior literature. Incomplete revascularization was documented if one or more angiographically relevant targets were not grafted. Although the ESC/EACTS Guidelines highlight the importance of completeness, they do not specify numerical thresholds.

### 2.5. Statistical Analysis

Statistical analyses, including propensity score matching (PSM) and standardized mean difference (SMD) calculations, and Kaplan–Meier survival analysis, were conducted using SPSS (Version 28.0, IBM Corp., Armonk, NY, USA). For graphical visualization of covariate balance, a Love Plot was generated using Python (Version 3.11, Python Software Foundation). An SMD < 0.15 was considered indicative of acceptable covariate balance.

The primary outcomes, including the incidence and severity of postoperative complications, were compared using the chi-squared (χ^2^) test for categorical variables and t-tests or Wilcoxon rank-sum test for continuous variables, depending on data distribution. Normality of continuous variables was assessed using the Shapiro–Wilk test. Normally distributed variables are presented as mean ± standard deviation (SD) and compared with Student’s *t*-test (two groups) or one-way ANOVA (more than two groups). Non-normally distributed variables are reported as median (interquartile range, IQR) and compared with the Mann–Whitney U test. For ANOVA analyses, post hoc pairwise comparisons with Bonferroni correction were applied where appropriate. Cox-regression, was applied for time-to-event data [10]. Sensitivity analyses, including ROC curve analysis, were conducted to assess the robustness of the findings. To assess the extent of multicollinearity among predictor variables in our regression model, we calculated the Variance Inflation Factor (VIF) for each variable [11] (threshold VIF > 10), guiding potential model adjustments. Candidate confounding factors for the multivariate model were selected based on clinical reasoning and those predictors that showed a *p*-value of ≤0.20 with the combined outcome in the univariable binary logistic regression model. Both crude and adjusted odds-ratios (OR) along with their 95% confidence intervals (CI) were calculated.

Multiple testing of secondary outcomes was addressed using Holm adjustment, with adjusted *p*-values presented in Supplementary Table S2. Two-tailed *p* ≤ 0.05 was considered statistically significant.

### 2.6. Propensity Score Estimation

In this study, propensity scores were estimated to adjust for potential confounders when comparing TAR with NTAR revascularization among the patient population described. Before matching, baseline demographics were analyzed using the χ^2^ test for categorical data and the Mann–Whitney-U test for continuous data. A logistic regression model was utilized, incorporating pre-operative demographic and clinical characteristics such as age, gender, body mass index (BMI), preoperative left ventricular ejection fraction (LVEF), Society of Thoracic Surgeons (STS) Score, EuroScore II, presence of left main disease, three-vessel disease, diabetes mellitus, smoking history, hypertension, chronic obstructive pulmonary disease (COPD), hyperlipidemia, history of stroke, and the surgical method (either On- or Off-pump).

### 2.7. Matching Procedure

Propensity scores were estimated via binary logistic regression including preoperative demographic and clinical covariates: age, gender, body mass index (BMI), preoperative LVEF, STS Score, EuroScore II, presence of left main or three-vessel disease, diabetes mellitus, smoking history, hypertension, chronic obstructive pulmonary disease (COPD), hyperlipidemia, prior stroke, and surgical method (on- vs. off-pump).

1:1 nearest-neighbor propensity score matching without replacement was performed using a caliper width of 0.02 (2% of the standard deviation of the logit of the propensity score) in SPSS (Version 28.0, IBM Corp., Armonk, NY, USA). Cohort balance was evaluated using standardized mean differences (SMD), with SMD < 0.15 considered indicative of acceptable covariate balance.

A Love Plot was generated in Python (Version 3.11) to visualize covariate balance, and exact SMD values before and after matching are provided in Supplementary Table S3. This study was reported in accordance with the STROBE (Strengthening the Reporting of Observational Studies in Epidemiology) guidelines.

## 3. Results

### 3.1. Preoperative Demographic and Clinical Characteristics Following Propensity Score Matching

**Figure 2:**
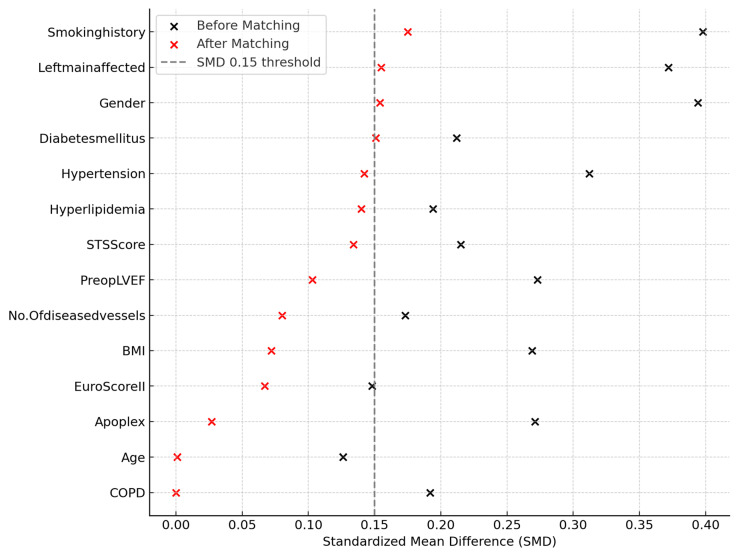
Covariate Balance Before and After Propensity Score Matching (Love Plot). Love Plot demonstrating standardized mean differences (SMD) for key baseline covariates before and after propensity score matching (TAR vs. NTAR). The vertical dashed line indicates the threshold for acceptable balance (SMD < 0.15).

In our retrospective multicenter study, we analyzed data from 240 heart failure patients with reduced ejection fraction (HFrEF) who underwent isolated coronary artery bypass grafting (CABG). After propensity score matching, patients were equally divided into two groups of 120 each: ‘Total Arterial Revascularization’ (TAR) and ‘Non-Total Arterial Revascularization’ (NTAR). Preoperative demographic and clinical characteristics (summarized in Table 1) showed remarkable similarity between both groups, with the only notable difference being a numerically higher proportion of elective surgeries in the NTAR group. Covariate balance after matching is illustrated in Figure 2 (Love Plot), and exact standardized mean differences (SMD) before and after matching are presented in Supplementary Table S3.

### 3.2. Primary Outcomes

**Figure 3:**
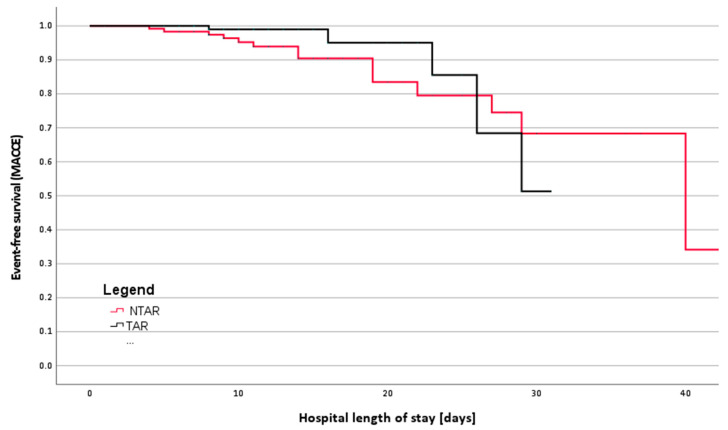
In-Hospital Event-Free Survival (MACCE) in TAR vs. NTAR. Kaplan–Meier curves illustrating in-hospital freedom from major adverse cardiac and cerebrovascular events (MACCE) in TAR (black) versus NTAR (red) patients. Censored observations were omitted for graphical clarity but included in the analysis. Log-rank *p* = 0.049.

In our retrospective study, operative and postoperative outcomes for propensity score matched cohorts are outlined in Table 2 and Table 3, with odds detailed in Table 4. Major adverse cardiac and cerebral events (MACCE) were significantly more frequent in the Non-Total Arterial Revascularization (NTAR) group at 14.2%, compared to 6.7% in the Total Arterial Revascularization (TAR) group, with a significant *p*-value of 0.007. Odds Ratio (OR) and Hazard Ratio (HR) for MACCE were notably lower in the TAR group (OR 0.643, CI 0.094–0.739, *p* = 0.011; HR 0.606, CI 0.213–1.727, *p* = 0.049). Kaplan–Meier survival analysis demonstrated a clear separation between groups (Figure 3), and ROC curve analysis showed good model predictive ability with an AUC of 0.750.

MACCE rates were consistently lower in the TAR group compared with NTAR in both OPCAB (3.2% vs. 15.6%) and ONCAB (7.4% vs. 12.5%). In logistic regression, TAR was associated with a significantly lower risk of MACCE (OR 0.18, 95% CI 0.04–0.68, *p* = 0.012), while the TAR × pump interaction was not significant (*p* = 0.29), indicating no heterogeneity by pump strategy (Supplementary Table S4).

In subgroup analyses, elective versus non-elective CABG procedures showed no significant differences. Mortality was numerically higher in NTAR patients (5.8% vs. 1.7% for TAR; *p* = 0.086), without statistical significance. Postoperative myocardial infarction was numerically more frequent in NTAR (5.0% vs. 3.3% in TAR; *p* = 0.333), and stroke occurred in 4.2% vs. 1.7%, respectively (*p* = 0.446).

Kaplan–Meier analysis demonstrated a lower in-hospital incidence of major adverse cardiac and cerebrovascular events (MACCE) in TAR patients compared to NTAR patients (log-rank *p* = 0.049). Event-free survival separated early during the postoperative course, with TAR maintaining higher MACCE-free survival throughout hospitalization (Figure 3).

### 3.3. Secondary Outcomes

The incidence of extracorporeal life support (ECLS) usage and acute kidney injury (AKI, KDIGO criteria [12]) did not differ significantly between groups (ECLS: 5.0% NTAR vs. 1.7% TAR; AKI: 10.9% in both groups). Dialysis requirement was numerically higher in TAR (10.0% vs. 6.7%), but not statistically significant.

NTAR patients required significantly more perioperative resources:

- ICU stay: median 90 h vs. 44.5 h (*p* < 0.001);
- Hospital stay: median 12 d vs. 10 d (*p* = 0.002);
- Ventilation time: median 12 h vs. 8 h (*p* < 0.001).

Postoperative delirium was more frequent in NTAR (14.2% vs. 5.0%; *p* = 0.016), corresponding to a 68% lower likelihood in TAR. Red blood cell (RBC) transfusions were also higher in NTAR:

- Intraoperative: 0.31 ± 0.58 units vs. 0.13 ± 0.45 units (*p* = 0.028);
- Entire hospital stay: 1.77 ± 2.91 units vs. 0.70 ± 1.33 units (*p* < 0.001).

Platelet (*p* = 0.902) and fresh-frozen plasma transfusions (*p* = 0.429) did not differ significantly.

After Holm adjustment for multiple comparisons, TAR remained associated with significantly lower delirium incidence and shorter ICU/hospital stays (Supplementary Table S2).

Complete revascularization was achieved in 81.7% of NTAR and 71.7% of TAR patients (*p* = 0.067). Median operative time from incision to closure did not differ significantly (208 min NTAR vs. 198.5 min TAR; *p* = 0.262). Resuscitation rates (2.5% vs. 1.7%), re-sternotomy for bleeding (1.7% each), and sepsis (5.0% vs. 2.5%) were also comparable between NTAR and TAR patients. Within the TAR cohort (n = 120), 55.0% received BIMA (± RA) and 33.3% LIMA + RA, with the remainder undergoing other arterial configurations. MACCE rates were comparable between BIMA (± RA) and LIMA + RA (4.5% vs. 5.0%; Fisher’s exact *p* = 1.00), suggesting that short-term outcomes within TAR were not driven by the specific arterial configuration (Supplementary Table S5). Moderate-to-severe MR (grade ≥2) did not differ between groups (TAR 21.7% vs. NTAR 25.8%; *p* = 0.519) and did not confound the association between TAR and MACCE (Supplementary Table S6).

## 4. Discussion

This study is clinically significant as it concentrates on a specific group—patients with heart failure and reduced ejection fraction (HFrEF) undergoing isolated Coronary Artery Bypass Grafting (CABG). It particularly highlights Total Arterial Revascularization (TAR), which literature suggests can yield better outcomes than traditional methods, despite its lower prevalence globally [13,14,15,16]. Employing multicenter, retrospective analysis with propensity score matching, the study provides insights into TAR’s efficacy, addressing selection bias typical in observational studies and enhancing the comparison between TAR and non-total arterial approaches. While our methodology accounted for center-level clustering, minor differences in perioperative management and surgical techniques remain a potential source of variability. Prospective studies with standardized protocols are needed to confirm and extend these findings.

TAR was associated with a reduced incidence of major adverse cardiac and cerebrovascular events (MACCE), supporting broader literature [3,17]. It should be noted that statistical significance was reached only for the composite MACCE endpoint, while individual components such as mortality, perioperative myocardial infarction, and stroke did not differ significantly between groups. This finding may reflect the limited power for single endpoints due to the low event rates and underscores that our results should be interpreted as hypothesis-generating rather than definitive proof of superiority of TAR in each individual outcome.

Our subgroup analysis found that patients undergoing urgent CABG were more frequently treated with TAR (61.6% vs. 38.4%, *p* = 0.007). Stratified analyses by on- vs. off-pump approach showed no effect modification, underscoring the robustness of the TAR association. Because both on-pump and off-pump CABG procedures were included, we evaluated potential heterogeneity by pump strategy. Stratified and interaction analyses did not indicate effect modification, supporting the robustness of the observed association between TAR and improved outcomes across surgical approaches. The lack of notable disparity in the wider elective versus non-elective cohort comparison could stem from diverse patient demographics and the varying degrees of clinical urgency, potentially masking the direct impact on outcomes across these groups. Nonetheless, the stark preference for TAR observed among the urgent patient subset accentuates the critical evaluation of TAR, suggesting its advantageous or preferred utilization in instances necessitating immediate surgical intervention. This observation necessitates a deeper exploration into the factors influencing the decision for TAR, particularly in settings where the expertise and operational capacity of the center and surgical team facilitate such a choice. This finding likely reflects institutional expertise and surgeon preference, as TAR was established as the default strategy in several participating centers, even in urgent cases. In addition, parallel conduit harvesting techniques may mitigate concerns about prolonged operative time, explaining why urgent patients in our cohort were not disadvantaged by TAR.

After PSM, in-hospital outcomes showed no significant difference between techniques. Risk prediction tools such as EuroSCORE II and STS-Score remain important but do not account for surgical strategy [18,19].

Research efforts to identify the optimal graft choice have led to numerous observational studies [3,20]. A landmark study by Sheikhy et al. in 2024 involving over a million patients demonstrated that multiple arterial grafting significantly enhances ten-year survival compared to single arterial grafting, with an adjusted hazard ratio of 0.87 [21]. It underscores the need for increased adoption of multiarterial grafting in clinical practice, despite current usage rates being low. These findings highlight the underutilized potential of multiarterial grafting and suggest upcoming shifts in clinical guidelines, pending outcomes from ongoing studies like the ROMA trial [22]. Contrastingly, the ART trial showed no significant difference in 10-year mortality (HR 0.96) or MACCE rates (HR 0.90) between bilateral and single internal mammary artery grafts. However, it noted a higher risk associated with saphenous vein grafts due to atherothrombosis, unlike arterial grafts that suffer from noncompetitive flow [20]. It is important to consider potential biases in observational studies, as surgeons’ preferences for certain surgical techniques could affect the reported outcomes.

In our study, increased in-hospital mortality in the NTAR group is consistent with meta-analyses showing TAR reduces both in-hospital and long-term mortality [13,23]. This consistency with prior research underscores the effectiveness of TAR, especially for patients with heart failure and reduced ejection fraction (HFrEF), marking it as a superior choice due to its impact on a critical outcome like mortality.

Additionally, while Extracorporeal Life Support (ECLS) trends towards more frequent use in NTAR, known for its crucial role in managing severe cardiac complications such as post-cardiotomy cardiogenic shock, this study was not focused on ECLS specifically. However, the necessity for meticulous patient selection is evident given the high mortality associated with ECLS and warrants further discussions about patient management strategies. Another significant advantage of TAR is its ability to minimize aortic manipulation, potentially reducing risks of postoperative complications such as stroke [24] and postoperative Delirium (POD) [25]. Although our study did not find a significant difference in the early incidence of stroke between TAR and NTAR groups, delirium was notably more prevalent in the NTAR group. The use of Propensity Score Matching (PSM) ensured a balanced distribution of on-pump and off-pump surgeries, which might explain the similarity in stroke occurrences.

While the long-term benefit of TAR is indeed linked to superior graft patency, our study identified additional mechanisms that may explain the observed early in-hospital benefits. Specifically, TAR avoids saphenous vein harvesting, which reduces surgical trauma and potential wound complications, and it minimizes aortic manipulation, thereby lowering the risk of perioperative embolic events. Furthermore, the exclusive use of arterial conduits is associated with lower transfusion requirements, likely due to reduced bleeding from fewer anastomoses on fragile venous grafts. These factors may collectively contribute to shorter ICU and hospital stays and lower rates of early complications observed in our TAR cohort.

Moreover, TAR was associated with decreased intraoperative and overall requirements for red blood cell (RBC) transfusions, supporting existing literature that TAR can significantly lower blood transfusion rates post-CABG [26]. But the relationship between RBC transfusions and patient outcomes in cardiac surgery is complex and controversial. Several studies have indicated negative outcomes associated with transfusing multiple units of RBCs, as it has been linked to severe infections and, in certain cases, increased mortality rates [27,28,29] and increased POD [30]. The debate on early extubation’s impact is noteworthy. While some authors see it as an indicator of patient severity, studies linking it to reduced hospital LOS [31], complement our observations on reduced ventilation times, advocating for TAR’s contribution to more efficient recovery protocols.

Within the TAR subgroup, outcomes did not differ between patients receiving BIMA (± RA) and those with LIMA + RA. MACCE rates were comparable (4.5% vs. 5.0%), and secondary outcomes including ICU and hospital length of stay, ventilation time, and operative duration showed no clinically relevant differences. These findings suggest that the survival and perioperative benefits of TAR in HFrEF patients are not driven by a specific arterial configuration, but rather by the exclusive use of arterial conduits. This aligns with prior reports indicating that both BIMA and LIMA + RA provide durable graft patency and favorable outcomes when compared with venous grafting [32].

Shorter ICU and hospital stays in TAR patients suggest faster recovery and improved outcomes [33]. This analysis hinted at a trend towards more complete revascularization in the NTAR group. Although completeness of revascularization was numerically lower in the TAR group (81.7% vs. 71.7%), this difference did not reach statistical significance. Importantly, sensitivity analyses (Supplementary Table S7) showed that completeness was not associated with early outcomes, suggesting that the observed benefits of TAR were not confounded by this factor [34,35].

While we did not collect data on deep sternal wound infections (DSWI), this represents a clinically relevant complication, particularly in patients undergoing BIMA grafting, and is recognized in current expert consensus as a potential drawback of multiple arterial grafting [36].

Future randomized trials are required to determine whether the observed early benefits of TAR—lower delirium rates, reduced transfusions, and shorter ICU stay—translate into improved long-term survival and neurocognitive outcomes in HFrEF patients.

## 5. Limitations and Future Perspective

This study has several limitations. First, its retrospective, multicenter design inherently limits causal inference, and although propensity score matching was applied to reduce selection bias, residual confounding from unmeasured variables (e.g., frailty, surgeon preference, or subtle intraoperative judgment) cannot be excluded. Second, despite attempts to harmonize perioperative management, inter-center variability in surgical techniques, transfusion thresholds, and ICU protocols may have influenced secondary outcomes. Third, follow-up was restricted to in-hospital results; therefore, graft patency, long-term survival, repeat revascularization, and neurocognitive recovery could not be assessed. Fourth, some variables, such as postoperative delirium and completeness of revascularization, were assessed retrospectively from institutional records, which may introduce classification bias. Finally, the low incidence of certain secondary endpoints (e.g., mortality, stroke) resulted in wide confidence intervals and limited statistical power. Furthermore, data on deep sternal wound infections (DSWI) were not available, precluding analysis of this important complication, which may particularly affect outcomes in patients receiving BIMA grafts.

Future prospective randomized trials with standardized perioperative protocols and extended follow-up are required to confirm whether the early benefits of total arterial revascularization observed in HFrEF patients translate into durable long-term survival and functional advantages. Furthermore, consistent data on guideline-directed medical therapy for HFrEF (e.g., beta-blockers, ACEi/ARNI, MRAs, SGLT2 inhibitors) were not available across all centers and therefore could not be analyzed. As our study focused on in-hospital surgical outcomes, we concentrated on perioperative variables that were uniformly documented. While propensity score matching minimized the risk of confounding, we cannot exclude residual influence of pharmacological therapy on outcomes.

## 6. Conclusions

This retrospective multicenter study suggests the benefits of total arterial revascularization (TAR) in patients with heart failure and reduced ejection fraction (HFrEF) undergoing coronary artery bypass grafting (CABG). TAR was associated with a reduced incidence of major adverse cardiac and cerebrovascular events (MACCE), shorter ventilation times, and reduced ICU and hospital length of stay, as well as a notably lower incidence of postoperative delirium—highlighting not only improved physical recovery but also mitigation of acute cognitive complications.

Importantly, statistical significance was observed for the composite MACCE endpoint, whereas individual components (mortality, perioperative myocardial infarction, stroke) did not differ significantly, likely reflecting the limited event rates in this cohort. These findings therefore warrant cautious interpretation.

Despite these encouraging results, the retrospective design, potential residual confounding despite propensity score matching, and the absence of long-term follow-up must be acknowledged. Moreover, variability in surgical techniques and perioperative care across centers may have influenced outcomes. Future prospective randomized trials with standardized protocols and extended follow-up are warranted to confirm these early benefits and support broader adoption of TAR in clinical practice for HFrEF patients.

## Figures and Tables

**Table 1:** Preoperative Demographic Data and Clinical Measurements.

Variable *n* (%)|mean (±SD)	Total Cohort	NTAR	TAR	*p* Value
	(*n* = 240)	(*n* = 120)	(*n* = 120)	
***Demographic Data***				
**Age**	67.11 (±9.59)	66.93 (±9.56)	67.30 (±9.65)	0.763 ^ANOVA^
**Gender**				
** Male**	221 (92.1%)	113 (94.2%)	108 (90.0%)	0.232 ^Chi2^
** Female**	19 (7.9%)	7 (5.8%)	12 (10.0%)	
**BMI**	28.09 (±4.68)	27.86 (±4.80)	28.32 (±4.57)	0.448 ^ANOVA^
***Clinical Measurements***				
**EuroScore II**	5.49 (±5.44)	5.62 (±5.52)	5.37 (±5.37)	0.718 ^ANOVA^
**STS Score**	3.01 (±2.50)	2.81 (±2.50)	3.21 (±2.49)	0.214 ^ANOVA^
**LVEF preop**	32.10 (±6.70)	31.82 (±6.15)	32.38 (±7.22)	0.519 ^ANOVA^
***Health Status***				
**Diabetes**				
** OAD**	58 (24.2%)	33 (27.5%)	25 (20.8%)	0.425 ^Chi2^
** Insulin dependent**	43 (17.9%)	22 (18.3%)	21 (17.5%)	
**Smoking history**				
** Former.**	66 (27.5%)	33 (27.5%)	33 (27.5%)	0.458 ^Chi2^
** Active.**	66 (27.5%)	37 (30.8%)	29 (24.2%)	
**Hypertension**	226 (94.2%)	115 (95.8%)	111 (92.5%)	0.271 ^Chi2^
**COPD**	54 (22.5%)	27 (22.5%)	27 (22.5%)	1.000 ^Chi2^
**Hyperlipidemia**	204 (85.0%)	105 (87.5%)	99 (82.5%)	0.278 ^Chi2^
**Apoplexy. pre-operative**	25 (10.4%)	13 (10.8%)	12 (10.0%)	0.833 ^Chi2^
**Carotid Stenosis**	37 (15.4%)	19 (15.8%)	18 (15.0%)	0.858 ^Chi2^
**Peripheral Vascular Disease**	64 (26.7%)	32 (26.7%)	32 (26.7%)	1.000 ^Chi2^
**Renal insufficiency**	86 (36.1%)	40 (33.3%)	46 (39.0%)	0.419 ^Fish^
***Cardiac Status***				
**Left main disease**	89 (37.1%)	39 (32.5%)	50 (41.7%)	0.142 ^Chi2^
**3-Vessel disease**	218 (90.8%)	109 (90.8%)	109 (90.8%)	1.000 ^Chi2^
** SR Rhythm pre-op**	187 (77.9%)	99 (82.5%)	88 (73.3%)	0.191 ^Chi2^
** NSTEMI**	70 (29.2%)	32 (26.7%)	38 (31.7%)	0.204 ^Chi2^
** STEMI**	20 (8.3%)	11 (9.1%)	9 (7.5%)	0.272 ^Fish^
**Previous PCI**	95 (39.6%)	46 (38.3%)	49 (40.8%)	0.692 ^Chi2^
**Type of surgery**				
** Elective**	117 (48.8%)	64 (54.7%)	53 (45.3%)	0.155 ^Chi2^
** Non-Elective**	123 (51.3%)	56 (45.5%)	67 (54.5%)	
**Off-pump**	120 (50.0%)	56 (46.6%)	64 (53.3%)	0.136 ^Chi2^

**Note:** Values are presented as mean ± SD or n *(%).* Abbreviations: ANOVA = analysis of variance; Chi2 = Chi-squared test; Fish = Fisher’s exact test; TAR = total arterial revascularization; NTAR = non-total arterial revascularization; SD = standard deviation; BMI = body mass index; STS = Society of Thoracic Surgeons; LVEF = left ventricular ejection fraction; OAD = obstructive airway disease; COPD = chronic obstructive pulmonary disease; PCI = percutaneous coronary intervention.

**Table 2:** Intra- and postoperative outcomes.

Variable	Total Cohort	NTAR	TAR	*p* Value
	(*n* = 240)	(*n* = 120)	(*n* = 120)	
***Intraoperative Requirement for Transfusion [Units]. mean (±SD)***				
**Packed red blood cells**	0.22 (±0.65)	0.31 (±0.58)	0.13 (±0.45)	0.028 ^ANOVA^
**Pooled thrombocytes**	0.16 (±0.52)	0.17 (±0.57)	0.16 (±0.46)	0.902 ^ANOVA^
**Fresh-frozen-plasma**	0.18 (±0.89)	0.23 (±1.04)	0.13 (±0.72)	0.429 ^ANOVA^
***Postoperative (after chest closure) Vasopressor and Inotropic requirements [γ]. mean (95% CI)***				
**Epinephrine**	0.015 (0.01–0.02)	0.018 (0.01–0.03)	0.01 (0.005–0.02)	0.008 ^MW^
**Norepinephrine**	0.12 (0.01–0.13)	0.17 (0.09–0.14)	0.10 (0.10–0.13)	0.028 ^MW^
***Postoperative Parameters. median (IQR)***				
**Hospital LOS (d)**	11 (9–16)	12 (9–18)	10 (8–14)	0.002 ^MW^
**ICU LOS (h)**	70 (31.75–120)	90 (48–143)	44.5 (22–104.75)	<0.001 ^MW^
**Ventilation (h)**	11 (5–18)	12 (6.25–24)	8 (5–15)	<0.001 ^MW^
**Surgical time (min)**	203.5 (166–246.75)	208 (166.5–259)	198.5 (165.25–236.5)	0.262 ^MW^
**Complete Revascularization. n (%)**	184 (76.7%)	98 (81.7%)	86 (71.7%)	0.067 ^Chi2^
***Transfusion requirements during the entire clinical stay [Units]. mean (±SD)***				
**Packed red blood cells**	1.23 (±2.38)	1.77 (±2.91)	0.70 (±1.33)	<0.001 ^ANOVA^
**Pooled thrombocytes**	0.25 (±0.77)	0.28 (±0.87)	0.23 (±0.67)	0.561 ^ANOVA^
**Fresh-frozen-plasma**	0.28 (±1.10)	0.38 (±1.27)	0.18 (±0.89)	0.178 ^ANOVA^

**Note:** Values are presented as mean ± standard deviation, median [interquartile range], or n (%), as appropriate. *p*-values refer to unadjusted comparisons between TAR and NTAR groups using chi-square or Mann–Whitney U tests. Holm-adjusted *p*-values for secondary endpoints are provided in Supplementary Table S2. Abbreviations: ANOVA = analysis of variance; Chi2 = Chi squared test; MW = Mann–Whitney U test; TAR = total arterial revascularization; NTAR = non-total arterial revascularization; SD = standard deviation; CI = confidence intervals; IQR = interquartile range; LOS = length of stay.

**Table 3:** Postoperative complications.

Variable *n* (%)	Total Cohort	NTAR	TAR	*p* Value
	(*n* = 240)	(*n* = 120)	(*n* = 120)	
**Resuscitation**	5 (2.1%)	3 (2.5%)	2 (1.7%)	1.000 ^Fish^
**Resternotomy**	4 (1.7%)	2 (1.7%)	2 (1.7%)	1.000 ^Fish^
**ECLS**	8 (3.3%)	6 (5.0%)	2 (1.7%)	0.181 ^Fish^
**AKI**	29 (12.89%)	13 (10.9%)	16 (13.4%)	0.559 ^Fish^
**Dialysis**	20 (8.3%)	8 (6.7%)	12 (10.0%)	0.484 ^Fish^
**Delirium**	23 (9.6%)	17 (14.2%)	6 (5.0%)	0.016 ^Chi2^
**Sepsis**	9 (3.8%)	6 (5.0%)	3 (2.5%)	0.209 ^Fish^
**Stroke**	7 (2.9%)	5 (4.2%)	2 (1.7%)	0.446 ^Fish^
**Postoperative MI**	10 (4.2%)	6 (5.0%)	4 (3.33%)	0.333 ^Fish^
**Mortality**	9 (3.8%)	7 (5.8%)	2 (1.7%)	0.086 ^Chi2^
**MACCE**	22 (9.2%)	17 (14.2%)	5 (4.1%)	0.007 ^Chi2^

**Note: Values are presented as n (%). ***p*-values refer to unadjusted comparisons between TAR and NTAR groups using chi-square tests. Holm-adjusted *p*-values for secondary endpoints are provided in Supplementary Table S2. Abbreviations: Chi2 = Chi squared test; Fish = Fisher´s exact test: TAR = total arterial revascularization; NTAR = non-total arterial revascularization; MACCE = major adverse cardiac and cerebrovascular events; MI = myocardial infarction; AKI = acute kidney injury; ECLS = extracorporeal life support.

**Table 4:** Odds and 95% CI of complications categorized by TAR versus NTAR.

Variable	OR	95% CI	B	*p* Value (Wald Statistic)
**Resuscitation**	0.648	0.037–11.45	−0.433	0.767
**Resternotomy**	1.353	0.236–121.42	1.678	0.292
**ECLS**	0.209	0.024–1.822	−1.566	0.156
**AKI**	0.902	0.243–3.352	−0.104	0.877
**Dialysis**	1.369	0.975–55.694	1.997	0.103
**Delirium**	0.290	0.10–1.027	−1.139	0.049
**Sepsis**	0.180	0.017–1.881	−1.713	0.152
**Stroke**	0.143	0.302–51.472	1.372	0.295
**Postoperative MI**	0.531	0.502–22.095	1.203	0.213
**Mortality**	0.399	0.011–0.861	−2.313	0.036
**MACCE**	0.643	0.094–0.739	−1.334	0.011

**Note:** Odds ratios (OR) with corresponding 95% confidence intervals (CI) are derived from binary logistic regression comparing TAR versus NTAR. Negative B values indicate lower odds for the TAR group. *p*-values correspond to Wald statistics from the regression models and are unadjusted for multiple testing. These outcomes were considered exploratory, and no adjustment for multiple testing was applied. Abbreviations: OR = odds ratio; CI = confidence interval; ECLS = extracorporeal life support; AKI = acute kidney injury; MI = myocardial infarction; MACCE = major adverse cardiac and cerebrovascular events.

## Data Availability

Data are contained within the article and the foundational research data can be made available upon request in compliance with the EU’s General Data Protection Regulation (GDPR). To ensure compliance, we will seek legal counsel in this matter.

## Supplementary Materials

The following supporting information can be downloaded at https://www.mdpi.com/article/10.3390/medsci13030179/s1, Supplementary Table S1: Institutional surgical and perioperative care protocols across participating centers. Supplementary Table S2: Holm-adjusted p-values for all secondary endpoints. Supplementary Table S3: Standardized mean differences (SMD) for baseline covariates before and after propensity score matching (TAR vs. NTAR). Supplementary Table S4: MACCE by TAR vs. NTAR stratified by Pump Strategy. Supplementary Table S5: Outcomes within TAR by arterial configuration. Supplementary Table S6: Mitral Regurgitation (MR) and Revascularization Strategy. Supplementary Table S7: Completeness of Revascularization and Short-term Outcomes.

## Abbreviations

AKI	acute kidney injury
ANOVA	analysis of variance
BIMA	bilateral internal mammary artery
CABG	coronary artery bypass grafting
CAM-ICU	Confusion Assessment Method for the Intensive Care Unit
CI	confidence interval
COPD	chronic obstructive pulmonary disease
ECLS	extracorporeal life support
EuroScore II	European System for Cardiac Operative Risk Evaluation II
Fish	Fisher´s exact test
HFrEF	heart failure with reduced ejection fraction
ICU	intensive care unit
IQR	interquartile range
LIMA	left internal mammary artery
LOS	length of stay
MACCE	major adverse cardiac and cerebrovascular events
MW	Mann-Whitney U test
NTAR	non-total arterial revascularization
ONCAB	on-pump coronary artery bypass
OPCAB	off-pump coronary artery bypass
OR	odds ratio
PSM	propensity score matching
RBC	red blood cell
SD	standard deviation
SMD	standardized mean difference
STS Score	Society of Thoracic Surgeons Predicted Risk of Mortality Score
TAR	total arterial revascularization
