# Supplementary material for: Total-Arterial Revascularization Is Superior in Heart Failure Patients with Reduced Ejection Fraction—A Propensity Score Matched Retrospective Multicenter Analysis
Source: Med Sci (Basel). 2025 Sep 5;13(3):179. doi: 10.3390/medsci13030179 (PMC12452580; doi:10.3390/medsci13030179)
Supplement: Supplementary file 1 [file medsci-13-00179-s001.zip › medsci-3826609-supplementary.pdf]

Supplementary Table S1: Institutional surgical and perioperative care protocols.

Center	Primary Graft Configuration (TAR)	Transfusion Threshold (Hb, g/dL)	Early Extubation Protocol	Delirium Screening Method
**1**	BIMA only	<8.0	Yes	CAM-ICU
**2**	BIMA only	<8.0	Yes	CAM-ICU
**3**	BIMA or LIMA + Radial Artery	<8.0	Yes	CAM-ICU
**4**	BIMA; Radial Artery (rarely)	<8.0	Yes	CAM-ICU

Abbreviations: TAR, total arterial revascularization; BIMA, bilateral internal mammary arteries; LIMA, left internal mammary artery; CAM-ICU, Confusion Assessment Method for the Intensive Care Unit; Hb, hemoglobin.

Supplementary Table S2: Holm-adjusted p-values for secondary endpoints.

Endpoint	Raw p-value	Holm-adjusted p-value
**Delirium**	0.016	0.032
**Hospital LOS (days)**	0.002	0.032
**ICU LOS (hours)**	0.001	0.032
**Ventilation time (hours)**	0.001	0.032
**RBC transfusion (entire stay)**	0.001	0.032
**Intraoperative RBC transfusion**	0.028	0.028

Note: Holm-adjustment was performed to account for multiple hypothesis testing of secondary endpoints. P-values < 0.05 after Holm adjustment were considered statistically significant.

Supplementary Table S3. Standardized mean differences (SMD) for baseline covariates before and after propensity score matching (TAR vs. NTAR).

Covariate	SMD Before PSM	SMD After PSM
**Smoking history**	0.398	0.175
**Left main affected**	0.372	0.155
**Gender**	0.394	0.154
**Diabetes mellitus**	0.212	0.151
**Hypertension**	0.312	0.142
**Hyperlipidemia**	0.194	0.140
**STS Score**	0.215	0.134
**Preop LVEF**	0.273	0.103
**No. of diseased vessels**	0.173	0.080
**BMI**	0.269	0.072
**EuroScore II**	0.148	0.067
**Apoplex**	0.271	0.027
**Age**	0.126	0.001
**COPD**	0.192	0.000

Note: An SMD < 0.15 was considered indicative of acceptable covariate balance.

Supplementary Table S4. MACCE by TAR vs. NTAR stratified by Pump Strategy.

Pump strategy	MACCE TAR (%)	MACCE NTAR (%)
**OPCAB**	3/93 (3.2%)	10/64 (15.6%)
**ONCAB**	2/27 (7.4%)	7/56 (12.5%)

Note. *Incidence of in-hospital MACCE in patients undergoing total arterial revascularization (TAR) versus non-total arterial revascularization (NTAR), stratified by pump strategy (ONCAB vs. OPCAB). A TAR × pump interaction test was not significant (p = 0.29).*

Supplementary Table S5: Outcomes within TAR by arterial configuration.

TAR configuration	n	MACCE n/N (%)	ICU LOS (h), median (IQR)	Hospital LOS (d), median (IQR)	Ventilation (h), median (IQR)	Operative time (min), median (IQR)
**BIMA (± RA)**	66	3/66 (4.5%)	28.5 (21.0–69.5)	10.0 (8.0–16.0)	8.0 (5.0–15.0)	200 (180–225)
**LIMA + RA**	40	2/40 (5.0%)	94.5 (45.8–130.5)	10.0 (8.8–12.0)	6.5 (4.0–15.8)	197 (175–220)

Note. Within the TAR cohort, outcomes were compared between BIMA (± radial artery) and LIMA + radial artery. MACCE rates were comparable (Fisher's exact test p = 1.00). Median ICU and hospital length of stay, and ventilation time are shown as median (IQR).

Supplementary Table S6. Mitral Regurgitation (MR) and Revascularization Strategy

S6A. Distribution of MR grades (0–3) by revascularization strategy (TAR vs. NTAR).

MR Grade	TAR (n, %)	NTAR (n, %)	Total (n, %)	p-value (Chi-square)
**0.0**	61 (50.8%)	36 (30.0%)	97 (40.9%)	
**1.0**	32 (26.7%)	51 (42.5%)	83 (35.0%)	
**2.0**	20 (16.7%)	30 (25.0%)	50 (21.1%)	
**3.0**	6 (5.0%)	1 (0.8%)	7 (3.0%)	
**Total**	119	118	237	0.001

Note: MR data missing in 3 patients.

S6B. Moderate-to-severe MR (grade ≥2) by revascularization strategy (TAR vs. NTAR).

MR Category	TAR (n, %)	NTAR (n, %)	p-value (Chi-square)
**Moderate–severe (≥2)**	26 (21.7%)	31 (25.8%)	0.519
**None–mild (0–1)**	93 (77.5%)	87 (72.5%)	

S6C. Logistic regression for in-hospital MACCE including TAR and moderate-to-severe MR (grade ≥2).

Predictor	Adjusted OR	95% CI (lower)	95% CI (upper)	p-value
**TAR (vs NTAR)**	0.26	0.09	0.74	0.011
**MR ≥2 (vs 0–1)**	1.13	0.41	3.08	0.817

Supplementary Table S7. Completeness of Revascularization and Short-term Outcomes.

Completeness of Revascularization	N	MACCE n/N (%)	ICU LOS (h), median (IQR)	Hospital LOS (d), median (IQR)
**Complete**	165	10/165 (6.1%)	36 (22–80)	10 (8–15)
**Incomplete**	75	5/75 (6.7%)	38 (24–85)	11 (8–16)

Note: Completeness of revascularization was assessed based on surgical reports and angiographic planning. Differences between groups may reflect anatomical limitations (e.g., diffuse or distal disease) or intraoperative judgment rather than technical failure. No significant association between incomplete revascularization and MACCE was observed.
